# Implementation strategies, facilitators, and barriers to scaling up and sustaining post pregnancy family planning, a mixed-methods systematic review

**DOI:** 10.1186/s12905-023-02518-6

**Published:** 2023-07-19

**Authors:** Ashraf Nabhan, Rita Kabra, Nahed Allam, Eman Ibrahim, Norhan Abd-Elmonem, Nouran Wagih, Nourhan Mostafa, James Kiarie, Family Planning Research Collaborators, Ahmed Zenhom, Alyaa Ashraf, Amal Alshabrawy, Emry Atwa, Fatma Elghamry, Mai Abouelnaga, Mariam Kodsy, Marwa Elgendi, Marwa Snosi, Menna Kamel, Mohamed Salama, Nada Makram, Noha Sakna, Salma Eltayeb, Samhaa bahnasy, Sara Galal, Shorouk Taha

**Affiliations:** 1 Department of Obstetrics and Gynecology, Faculty of Medicine, Ain Shams University, Ramses Street, Cairo, Egypt; 2 Department of Sexual and Reproductive Health Including UNDP/UNFPA/ UNICEF/WHO/World Bank Special Programme of Research, Development and Research Training in Human Reproduction, World Health Organization, Geneva, Switzerland; 3 Department of Obstetrics and Gynecology, Faculty of Medicine, Al Azhar University, Cairo, Egypt; 4 Kafrelsheikh University, Kafr el-Sheikh, Egypt; 5 Faculty of Medicine, Ain Shams University, Cairo, Egypt

**Keywords:** Family planning, Post-abortion, Postpartum, Contraception, Scaling-up

## Abstract

**Background:**

Post pregnancy family planning includes both postpartum and post-abortion periods. Post pregnancy women remain one of the most vulnerable groups with high unmet need for family planning. This review aimed to describe and assess the quality of the evidence on implementation strategies, facilitators, and barriers to scaling up and sustaining post pregnancy family planning.

**Methods:**

Electronic bibliographic databases (MEDLINE, PubMed, Scopus, the Cochrane Library, and Global Index Medicus) were searched from inception to October 2022 for primary quantitative, qualitative, and mixed method reports on scaling up post pregnancy family planning. Abstracts, titles, and full-text papers were assessed according to the inclusion criteria to select studies regardless of country, language, publication status, or methodological limitations. Data were extracted and methodological quality assessed using the Mixed Methods Appraisal Tool. The convergent integrated approach and a deductive thematic synthesis were used to identify themes and sub-themes of strategies to scale up post pregnancy family planning. The health system building blocks were used to summarize barriers and facilitators. GRADE-CERQual was used to assess our confidence in the findings.

**Results:**

Twenty-nine reports (published 2005–2022) were included: 19 quantitative, 7 qualitative, and 3 mixed methods. Seven were from high-income countries, and twenty-two from LMIC settings. Sixty percent of studies had an unclear risk of bias. The included reports used either separate or bundled strategies for scaling-up post pregnancy family planning. These included strategies for healthcare infrastructure, policy and regulation, financing, human resource, and people at the point of care. Strategies that target the point of care (women and / or their partners) contributed to 89.66% (26/29) of the reports either independently or as part of a bundle. Point of care strategies increase adoption and coverage of post pregnancy contraceptive methods.

**Conclusion:**

Post pregnancy family planning scaling up strategies, representing a range of styles and settings, were associated with improved post pregnancy contraceptive use. Factors that influence the success of implementing these strategies include issues related to counselling, integration in postnatal or post-abortion care, and religious and social norms.

**Trial registration:**

Center for Open Science, OSF.IO/EDAKM

**Supplementary Information:**

The online version contains supplementary material available at 10.1186/s12905-023-02518-6.

## Abstract (plain language summary)

Family planning could prevent one third of maternal deaths by allowing women to delay motherhood, avoid unintended pregnancies and subsequent abortions. Post pregnancy family planning includes both postpartum and post-abortion periods. Post pregnancy women remain one of the most vulnerable groups with high unmet need for family planning. Scaling up post pregnancy family planning is essential for achieving universal access to reproductive health-care services. Reports of strategies to scale up post pregnancy family planning were systematically reviewed and summarized. These included strategies for healthcare infrastructure, policy and regulation, financing, human resource, and people at the point of care. Strategies that target the point of care (women and/or their partners) contributed to 89.66% (26/29) of the reports either independently or as part of a bundle. Point of care strategies increase adoption and coverage of post pregnancy contraceptive methods. Certain factors influence the success of implementing these strategies, including issues related to counselling, integration in postnatal or post-abortion care, and religious and social norms.

**Supplementary Information:**

The online version contains supplementary material available at 10.1186/s12905-023-02518-6.

## Background

Post pregnancy women have a high unmet need for family planning (FP). Post pregnancy family planning (PPFP) includes both postpartum and post-abortion periods. The World Health Organization (WHO) recommends spacing pregnancies by two years or more following the delivery of a newborn, and at least six months after receiving post-abortion care [1]. This recommendation is based on evidence that PPFP reduces the burden of maternal and perinatal adverse events [2].

Despite this, there are still major missed opportunities for FP among postpartum women in many low- and middle-income countries (LMIC) and many post-abortion clients still leave the facility without a contraceptive method [3, 4].

Therefore, scaling up PPFP is important to allow women to delay motherhood, avoid unintended pregnancies and subsequent abortions, and consequently preventing maternal morbidity and mortality [5, 6]. Investing in scaling up PPFP can accelerate achievement across Sustainable Development Goal [7].

Scaling up is defined as deliberate efforts to increase the impact of health service innovations successfully tested in pilot or experimental projects to benefit more people and to foster policy and program development on a lasting basis [8–10].

The WHO has commissioned this systematic review of scaling up of post pregnancy family planning. The overall aim of the review is to describe and assess the quality of the evidence on implementation strategies, facilitators, and barriers to scaling up and sustaining post pregnancy family planning. The review has the following objectives:

- to identify, appraise and synthesize research evidence regarding the approaches or strategies to scaling up PPFP for improving coverage and sustainability.
- to identify, appraise and synthesize research evidence on the barriers to and facilitators of scaling up of PPFP.

## Methods

This systematic review followed the JBI methodology for mixed methods systematic reviews (MMSR) [11] and methods suggested by the Cochrane Effective Practice and Organisation of Care (EPOC) Review Group [12]. The protocol, available as a preprint [13], was registered in the Center for Open Science platform (https://doi.org/10.17605/OSF.IO/EDAKM). The full review is reported according to the Preferred Reporting Items for Systematic Reviews and Meta-Analyses (PRISMA) [14].

### Criteria for considering studies for this review

#### Types of studies

Reports of primary studies, either quantitative, qualitative, process evaluation, policy analysis, and case studies were considered eligible. Mixed method studies were considered if data from the quantitative or qualitative components can be clearly extracted. Editorials, commentaries, proposals, conference abstracts and systematic reviews were excluded. Reports that lacked a clear methodology section were also excluded if clarification could not be obtained from the authors. There were no restrictions on length of study follow-up, language of publication, or country of origin.

#### Types of participants

Study participants were the targets of strategies that would scale up PPFP, whether individuals (recipients of care, providers of care, other stakeholders), organizations, or systems.

#### Types of scaling up strategies

Approaches or strategies of scaling-up [9, 15–17] healthcare infrastructure-related (e.g., providing medical equipment or changing linkages within a health system), policy and regulation-related (e.g., revising policy to allow widespread community-based case management of a disease), financing-related (e.g., changing payment mechanisms), human resource-related (e.g., training and deployment of health care providers, changing roles of administrators), and patient-related (e.g., involving patients/public in recruitment or promotion).

#### Types of outcome measures

Implementation research outcomes mainly adoption (the intention, initial decision, or action to try to employ a new intervention; also known as Uptake, Utilization, Intention to try), coverage (the degree to which the population that is eligible to benefit from an intervention actually receives it.), and sustainability (the extent to which an intervention is maintained or institutionalized in a given setting; also known as maintenance, continuation) [18, 19].

### Barriers to and facilitators (Factors that influence scaling up of PPFP)

The approach to the factors affecting scaling up was based on Supporting the Use of Research Evidence (SURE) framework [20], namely factors related to recipients of care, providers of care, other stakeholders (including other healthcare providers, community health committees, community leaders, program managers, donors, policymakers, and opinion leaders), health system constraints, and social and political constraints (Supplementary file 1).

Factors were grouped by the categories of health system building blocks (HSBB). HSBB is an analytical framework used by WHO to describe health systems, disaggregating them into 6 core components with the people in the center: (i) service delivery, (ii) health workforce, (iii) health information systems, (iv) Medical products, vaccines, and technologies (access to essential medicines), (v) financing, and (vi) leadership and governance [21].

### Literature search

#### Sources

Bibliographic databases were searched for peer reviewed publications as well as grey literature. We performed the search strategy to identify published studies in the following electronic bibliographic databases (from inception to October 2022): MEDLINE, PubMed, Scopus, the Cochrane Library, and Global Index Medicus, World Health Organization (www.globalindexmedicus.net). Search also included gray literature using the search engines and websites of relevant organizations. The reference list of all included reports was screened for additional studies.

#### Search strategy

The search terms were developed a priori. We followed recommendations of a previous review about terms to use for scaling up [22]. The search strategy was first developed in Pubmed format and was adapted to the other databases. The full search strategies for various platforms are available in an open access repository [23]. For unpublished studies, the review authors contacted global experts in family planning to identify possible reports. The email was sent through 3 major mailing lists maintained by relevant international organizations in the field of family planning.

The search strategies utilized the following terms (“Implementation Science” [MeSH Terms] OR scaling-up [Text Word] OR Scalability [Text Word] OR Scale-up [Text Word]) AND (“Family Planning Services” [MeSH Terms] OR contraception [MeSH Terms] OR contracept*[Text Word] OR “family planning” [Text Word]). The search aimed at sensitivity rather than precision since we opt to minimize false negative results.

#### Management of search results

All search results were imported into Jabref v5. Duplicate search results were identified by the software and were eliminated using a method that enables retaining unique citations without accidentally excluding false duplicates.

### Data collection

#### Study selection

After removal of duplicates, two review authors (EI, NA) independently piloted the study selection form with a small random sample of studies to assess understanding of eligibility criteria and ease of use of the form. Two review authors (NW, NM) independently screened all titles/abstracts and full text to identify the relevant studies. Discrepancies between review authors regarding study eligibility was resolved by consensus or, when required, with a third party (AN). PRISMA flowchart was used to describe the process of study selection.

#### Data extraction

Two review authors (NW, NM) used a data extraction form (Supplementary file 2) adapted from JBI Mixed Methods Data Extraction Form following a Convergent Integrated Approach [11], to independently extract characteristics from the included studies: study title, first author, year of publication, country of study, the country’s economic status (low-, middle-, or high-income), funding source, study setting, facility type, study type (qualitative, quantitative and mixed methods studies). Data extraction included the components of scaling-up strategies mentioned in each study, the target of the scale up activity, the time frame of the scaling-up process, implementation outcome evaluated in each study, and barriers and facilitators. Any disagreement in the data collection process was resolved through discussion and consensus between the two reviewers and, if needed, with a third party (AN).

### Quality assessment

For each included study, the methodological quality was described using the corresponding Mixed-Methods Appraisal Tool (MMAT) criteria (Supplementary file 3). [24, 25] Two independent reviewers (NW, NM) assessed the quality of included studies using MMAT, with a third independent reviewer (AN) to be used in case of any discrepancies. Studies were not excluded based on methodological limitations, but rather used to assess the confidence in the findings.

### Data synthesis

A convergent integrated approach was used. This involved transformation into textual descriptions or narrative interpretation of the quantitative results in a way that answers the review questions. Articles were first grouped according to component(s) of scaling up, as defined above. A deductive thematic synthesis used the SURE framework and the health system building blocks to synthesize the factors affecting implementation (barriers and facilitators).

### Appraisal of confidence in the review findings

GRADE‐CERQual was used to assess the confidence that can be placed in each review finding [26]. GRADE‐CERQual approach assesses confidence in the evidence based on four components: methodological limitations of included studies, coherence of the review findings, adequacy of the data contributing to a review finding, and relevance of the included studies to the review question.

After assessing each of the four components, a judgement about the overall confidence in the evidence supporting each review finding was made. The judgment of confidence was either high, moderate, low, or very low. The final assessment was based on consensus among the review authors. Summaries of the findings and the assessments of confidence in these findings were presented in Tables 3 and 4.

### Researchers’ reflexivity

We maintained a reflexive stance throughout the stages of the review process, from study selection to data synthesis, as detailed in the review protocol [13].

## Results

### Study selection

**Fig. 1:**
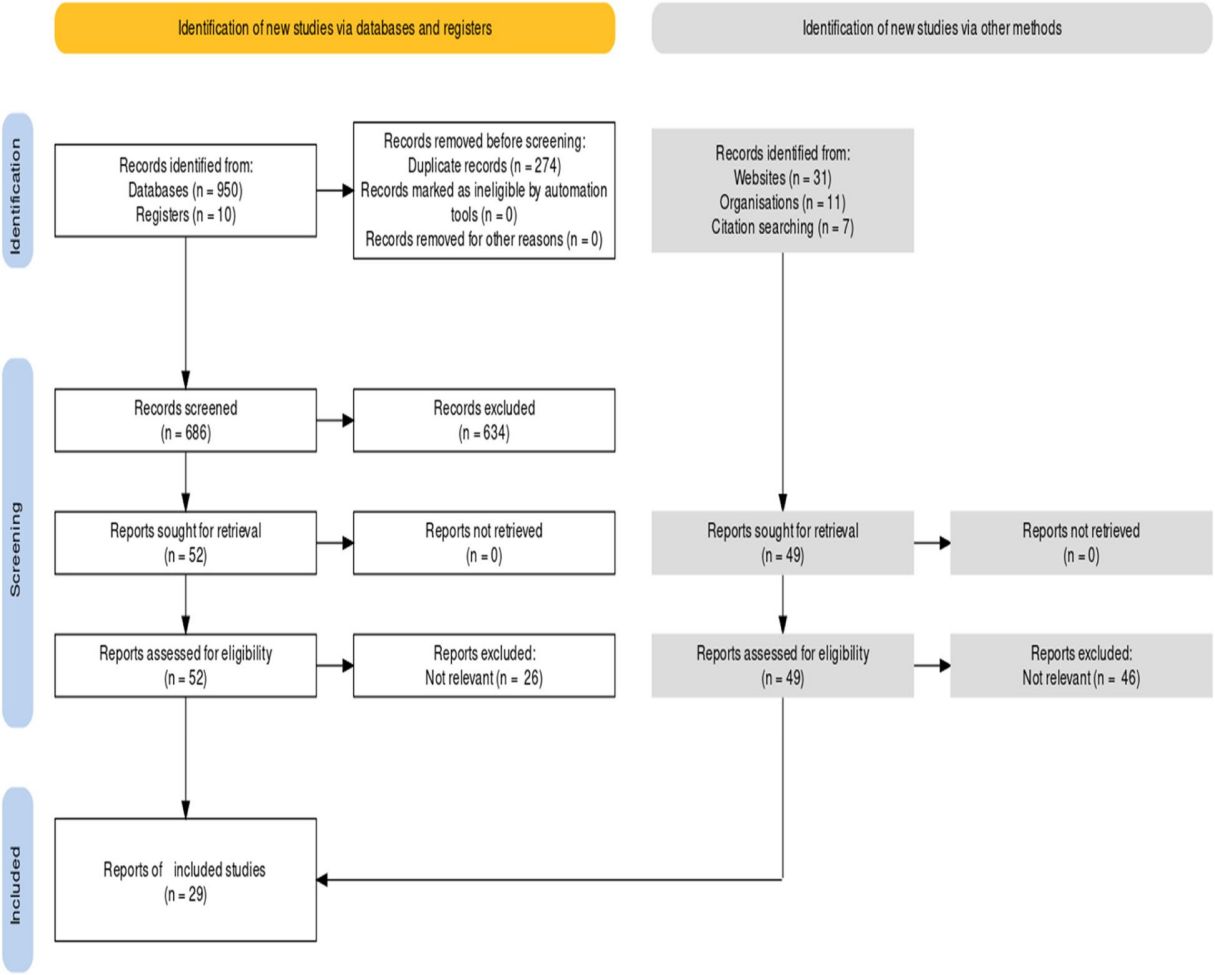
PRISMA Flowchart

The flow of identification, screening, and including 29 reports is depicted in Fig. 1

### Findings of the review

#### Characteristics of included studies

The 29 included studies [27–55] (Table 1) used quantitative (19/29; 65.52%), qualitative (7/29; 24.14%), and mixed methods (3/29; 10.34%). The studies were all published between 2005 and 2022. The studies werereported from 37 countries, from all regions, and from LMIC and High-income countries. Eight studies were reported from the USA [27–34]; four from Tanzania [35–38], two from Sri Lanka [39, 40], Nigeria [41, 42], Nepal [43, 44], Rwanda [45, 46], Bangladesh [38, 47], and one study from Benin [48], Bolivia [49], Burkina Faso [50], Chad [48], Côte d’Ivoire [48], Democratic Republic of Congo [50], Guatemala [51], India [52], Liberia [53], Mexico [49], Niger [48], Pakistan [54], Senegal [48], Togo [48], and Turkey [55].

**Table 1:** Characteristics of included studies

**Author-Year**	**Country**	**Type of study**	**Design**	**Sample**	**Scaling up category**
Akman 2010 [55]	Turkey	Quantitative	RCT	180	Recipient of care
Billings 2007 [49]	Bolivia, Mexico	Qualitative	IDI	49	Healthcare infrastructure, Policy and regulation, Financing, Human resource, Recipient of care
Cooper 2014 [47]	Bangladesh	Qualitative	IDI, FGD	40	Recipient of care
deSilva 2021 [39]	Sri Lanka	Qualitative	IDI	12	Healthcare infrastructure, Policy and regulation, Financing, Human resource, Recipient of care
DeSisto 2019 [27]	USA	Qualitative	IDI	41	Healthcare infrastructure, Policy and regulation, Financing, Human resource, Recipient of care
Eluwa 2016 [41]	Nigeria	Quantitative	BA	728	Recipient of care
Espey 2021 [45]	Rwanda	Quantitative	BA	12,068	Human resource, Recipient of care
Ingabire 2018 [46]	Rwanda	Quantitative	BA	9020	Human resource, Recipient of care
Karra 2019 [40]	Sri Lanka	Quantitative	stepped-wedge cluster RCT	39,084	Healthcare infrastructure, Human resource, Recipient of care
Kestler 2006 [51]	Guatemala	Quantitative	BA	13,928	Healthcare infrastructure, Human resource, Recipient of care
Koch 2022 [28]	United States	Quantitative	Retrospective Cohort	6233	Financing
Lacy 2020 [29]	United States	Quantitative	QI	2012	Healthcare infrastructure, Policy and regulation, Financing, Human resource, Recipient of care
Nelson 2019 [53]	Liberia	Mixed methods	FGD, KII, CBA	1066	Policy and regulation, Human resource, Recipient of care
Palm 2020 [30]	United States	Mixed methods	IDI, stepped wedge	20	Healthcare infrastructure, Policy and regulation, Financing, Human resource, Recipient of care
Pearson 2020 [35]	Tanzania	Quantitative	Stepped-wedge cluster RCT	15,264	Healthcare infrastructure, Human resource, Recipient of care
Pleah 2016 [48]	Benin, Chad, Côte d’Ivoire, Niger, Senegal, Togo	Quantitative	BA	15,000	Human resource
Pradhan 2019 [43]	Nepal	Quantitative	Stepped wedge RCT	75,587	Healthcare infrastructure, Human resource, Recipient of care
Rasch 2005 [36]	Tanzania	Qualitative	cross-sectional	1365	Human resource, Recipient of care
Saeed 2008 [54]	Pakistan	Quantitative	RCT	600	Recipient of care
Sebastian 2012 [52]	India	Quantitative	RCT	959	Recipient of care
Simmons 2013 [31]	USA	Quantitative	RCT	50	Financing, Recipient of care
Sodje 2016 [42]	Nigeria	Quantitative	Prospective cohort	1061	Human resource, Recipient of care
Stephens 2019 [37]	Tanzania	Quantitative	cross-sectional	6636	Healthcare infrastructure, Policy and regulation, Financing, Human resource, Recipient of care
Tang 2014 [32]	USA	Quantitative	RCT	800	Recipient of care
Tran 2018 [50]	Burkina Faso, Democratic Republic of Congo	Qualitative	IDI, FGD	213	Healthcare infrastructure, Policy and regulation, Financing, Human resource, Recipient of care
Wilkinson 2019 [33]	United States	Quantitative	Retrospective Cohort	1072	Financing
Wu 2020 [44]	Nepal	Mixed methods	BA	953	Recipient of care
Yahner 2022 [38]	Bangladesh, Tanzania	Qualitative	IDI, FGD	60	Human resource, Recipient of care
Zerden 2015 [34]	United States	Quantitative	RCT	324	Recipient of care

*BA* Before after, *CBA* controlled before after, *FGD* focus group discussion, *IDI* in-depth interview, *KII* key informant interviews, *RCT* randomized controlled trial

### Methodological quality

Most of the included reports (17/29; 58.62%) had unclear risk of bias, with 9/29 (31.03%) were judged to be at high risk if bias.

### Strategies of scaling-up post pregnancy family planning

The included 29 reports [27–55] described unique yet interrelated strategies of scaling-up post pregnancy family planning including healthcare infrastructure, policy and regulation, financing, human resource, and recipient of care. Most reports (19/29; 65.52%) utilized a combination of these strategies, Table 2.

**Table 2:** A matrix of reported scaling up strategies

**Component**	**Description**	**References**
Human resource		[27, 30, 35, 36, 41, 45, 46, 48, 50, 51, 53]
Training/continuing education of Health care providers	Training for clinicians, support staff, and administrative staff through various modalities (e.g., small-group in-person training, one-on-one proctoring, virtual Webinar series) on topics including family planning; medical management of contraception; hands-on clinical skills (e.g., Long-Acting Reversible Contraception (LARC) insertion and removal); billing, coding, and reimbursement; and preventing coercion and bias	
Ongoing technical assistance	Ongoing, targeted technical assistance to clinicians, support staff, and administrative staff through various modalities (e.g., coaching calls, in-clinic training specialists) on topics including hands-on clinical skills; purchasing, stocking, and billing for contraceptives; patient education materials; contraceptive access policies/procedures; contraceptive workflow; and data collection and reporting	
Financing		[36, 41, 46]
Provision of low- or no-cost contraception	Direct funding or stocking for participating health centers across delivery settings to offer contraceptive methods and services to eligible individuals at low or no cost	
Grants for equipment/supplies	Direct funding to health care facilities to purchase contraceptive supplies and equipment, other clinic supplies, and supplies for personnel Providing reimbursement to facilities for administrative cost, technical, and logistic control	
Health care facility level		[27, 35, 49–51, 53]
Package Service	Offering modern contraception as part of postnatal care (PNC) or post-abortion care (PAC) services Integration of PAC into existing health systems as a part of their regular service delivery FP and Immunization integration, intra-facility referrals between FP and vaccination	
	Improving access to technologies and equipment as manual vacuum aspiration (MVA), medications, pain Control and contraceptive methods Strengthening each hospital’s infrastructure for post-abortion care Instituting an abortion surveillance system and using it to increase provision of post-abortion care	
Quality improvement	Continuous quality improvement to identify barriers and potential strategies to address those barriers; ongoing measurement of aggregate, de-identified data on use of various contraceptives; provision of contraception services or person-centered counseling; and knowledge, skills, attitudes, or beliefs about contraception among providers	
Recipients of care		[27, 35, 36, 45–47, 50, 51, 54, 55]
Awareness campaign	Digital media and marketing campaigns to increase awareness about the availability of reproductive health services and provide information and resources on reproductive health topics	
Stakeholder engagement	Engagement in multi-stakeholder partnerships with public and private entities for effective implementation	
	Developing and distributing informational materials (Information education and communication (IEC) materials on PPFP, including leaflets and a video that played in the waiting room) Counseling sessions with postpartum women and group meetings with mothers-in-law, postpartum women, and men Fictional stories presented in leaflet and oral form within home visits and group discussion sessions Involving women in the promotions to improve understanding the importance of PPFP & postpartum intrauterine device (PPIUD) Reminder cards are given to women at each follow-up visit to remind them of the next visits. Cards would also contain a message to stress the health benefits of follow-up visits Counseling with more time allocated to specific topics Prenatal one to one counselling on postpartum contraception	
Policy and regulation		[27, 49]
Policy change	Overall public and private insurance coverage for contraception, such as LARC coverage and reimbursement and multiple months of dispensing; expanded ability of providers to prescribe and dispense contraception; ensured payment parity for providers; over-the-counter contraception without a prescription	

### Effect of strategies for scaling up post pregnancy family planning

Strategies that target the point of care (women and / or their partners) contributed to 89.66% (26/29) of the reports either independently (Table 3) or as part of a bundle (Table 4) to scale up post pregnancy FP. Point of care, financial, and health resources strategies improved adoption and coverage of post pregnancy contraceptive methods (moderate certainty evidence).

**Table 3:** Summary of the reports of unique post pregnancy family planning scaling up strategies

**Main theme**	**Outcomes**	**Number of studies**	**Summarized review finding**	**GRADE-CERQual Assessment**
Point of care	Adoption, Coverage	8	Point of care strategies increase the use of post pregnancy contraceptive methods	Moderate confidence
Financing	Adoption, Coverage	2	Financing strategies increase the use of post pregnancy contraceptive methods	Low confidence
Human resources	Adoption, Coverage	1	Human resource strategies increase the use of post pregnancy contraceptive methods	Low confidence

**Table 4:** Summary of the reports of multifaceted post pregnancy family planning scaling up strategies

**Main theme**	**Outcomes**	**Number of studies**	**Summarized review finding**	**GRADE-CERQual Assessment**
Healthcare infrastructure PLUS Policy and regulation PLUS Financing PLUS Human resource PLUS point of care	Adoption, Coverage	7	Healthcare infrastructure, Policy and regulation, Financing, Human resource, point of care: may increase the use of immediate postpartum long-acting reversible contraception	Moderate confidence
Human resource PLUS point of care	Adoption, Coverage	5	Human resource, point of care: increase the use of post pregnancy contraceptive methods	Moderate confidence
Healthcare infrastructure PLUS Human resources PLUS point of care	Adoption, Coverage	4	Healthcare infrastructure, Human resources, point of care: increase the use of post pregnancy contraceptive methods (Post abortion, Immediate PPIUD)	Moderate confidence
Financing plus point of care	Adoption, Coverage	1	Financing plus point of care: may increase the use of post pregnancy contraceptive methods	Very Low confidence
Policy and regulation PLUS Human resource PLUS point of care	Adoption, Coverage	1	Policy and regulation, Human resource, point of care: may increase the use of post pregnancy contraceptive methods	Very Low confidence

### Factors influencing scaling up of PPFP

The health system building blocks framework was used to allow synthesis of factors that influence the scaling up of PPFP, Table 5. The most notable barriers to scaling up PPFP included failure to provide effective counselling, lack of integration of PPFP in postnatal or post-abortion care, and negative religious and traditional norms.

**Table 5:** Factors that influence the scaling up of post pregnancy family planning

**Category**	**Factor**	**Reference**
People	Family involvement, accompaniment, and tradition	[38]
	Fear of judgment	[38]
	Lack of interest	[45]
	Knowledge regarding lactational amenorrhea and suitable contraceptive methods	[50]
	Loyalty toward the religious doctrines in religious based hospitals in post abortion contraceptive counselling instead of applying national family planning guidelines	[36]
	Male partner:	
	Integration of men	[45]
	Partner sharing in decision making	[47]
	Myths and misinformation, Misconceptions about modern contraception	[38, 50]
	Perceived quality of facility services	[38]
	Factors related to postnatal care	
	Prioritization by women of scheduled postpartum visits	[50]
	Opportunities to encourage continuity of care, especially for PPFP	[38]
	A contraception-dedicated six-week postpartum	[50]
	Religious and traditional norms:	
	Sexual abstinence for up to three to six months postpartum	[50]
	Social pressure to closely space pregnancies	[38]
	Traditional views on the consequences borne by closely spaced children and their mothers	[50]
	Cultural and religious objections to family planning and lingering misconceptions	[48]
Service delivery	Access to facility services	[38]
	Factors related to counselling	
	dedicated PPFP counseling materials	[50]
	privacy within the health facility	[53]
	time necessary to fully counsel women on all available and appropriate methods	[45]
	Time required for One-to-one counseling	[55]
	Limited availability of clinic days and scheduled visits dedicated to contraception	[50]
	Extent of antenatal care (ANC) coverage	[48]
Medical products	Available equipment and supplies	[48]
	Availability of readily accessible methods and plans for stock-outs in health facilities	[50]
Financing	Challenges with Engaging private insurance companies	[27]
	Financial risk intolerance	[30]
	LARC device cost/reimbursement	[27, 30]
	Administrative infrastructure and financial flexibility	[30]
	Out-of-pocket payment of contraceptives	[50]
	Cost/Fund to buy or to purchase the instruments or LARC by health facilities	[27, 49]
Health information systems	Challenges in acquiring data use agreements between public health and medicaid	[27]
	Difficulty analyzing raw medicaid claims data	[27]
	Long duration for resolving technical billing issues	[27]
	Technical complexity of information technology system for claims processing	[27]
	Pre-existing strong collaborations across agencies with respect to data	[27]
Leadership and Governance	Leadership stability	[30]
	Support from high-level leadership	[27]
	Clinical champions	[27, 30]
	Co-location of health department and financial agency and/or strong pre-existing working relationship between agencies	[27]
	Connecting with rural birthing facilities	[27]
	Translating what works across various contexts	[27]
	Effect of political sensitivity around contraception on team’s ability to work on increasing LARC access	[27]
	Political commitment to post abortion and postpartum FP programs	[49, 50]
	Process changes for coders and pharmacy staff members	[27]
Health workforce	Ability to work with other teams in the learning community and share resources	[27]
	Continued support and guidance from trainers in informal follow-up visits and phone calls	[48]
	Judgmental treatment from health providers	[38]
	Inability to perform the procedure or Lack of knowledge/skills about all contraceptive methods	[45, 48]
	Lack of live clinical insertions	[45]
	Lack of supervision throughout practice insertion sessions	[45]
	Pre-existing personal connections of team members	[27]
	Shared culture and language facilitated the training, reduced miscommunication between teams, and built engagement and mutual support	[48]
	Spill over: hearing about process from others in the learning community	[27]
	Team members long and continuous involvement with immediate postpartum LARC initiative	[27]
	Turnover in team members	[27]
	Uncertainty about goal for immediate postpartum contraceptive use	[27]

## Discussion

### Summary of the evidence

The review identified unique yet interrelated strategies of scaling-up post pregnancy family planning including healthcare infrastructure, policy and regulation, financing, human resource, and recipient of care. Most reports (19/29; 65.52%) utilized a combination of these strategies. Results show that point of care strategies, financing strategies, human resource strategies increase the use of post pregnancy contraceptive methods.

The review highlighted core components of strategies for scaling up post pregnancy family planning. The results agree with and update previously published reviews [56]. These components include training or continuing education and ongoing technical assistance at the health care provider level; provision of low- or no-cost contraception, grants for contraceptive equipment or supplies, and quality improvement and monitoring at the health facility level; public awareness campaigns and stakeholder engagement at the community level; and legislation or other policy changes at the public policy level. Implementation of these intervention components is interrelated and represents a theory-based, systems change approach wherein multiple interventions are implemented across levels to maximize effects across diverse and often fragmented systems of care in different countries.

The results of the current review agree with a previous review [57] that suggested that offering modern contraception services as part of care provided during childbirth or abortion increases post pregnancy contraceptive use and is likely to reduce both unintended pregnancies and pregnancies that are too closely spaced. Evidence for sustainability is insufficient and this remains an important issue to maintain a reduction in unmet needs for postpartum or post abortion periods. The need for integration with health system is critical for family planning to be institutionalized and therefore sustainable [58].

Improving the effectiveness of family planning programs is critical for empowering women and adolescent girls, improving human capital, reducing dependency ratios, reducing maternal and child mortality, and achieving demographic dividends particularly in low- and middle-income countries [59].

The current review critically summarized the factors that affect the success of scaling up of PPFP. The most apparent factors influencing the success of implementing these strategies include factors related to effective counselling and challenges in the integration of PPFP in postnatal or post-abortion care. These factors should be carefully considered by policymakers and family planning service planners in the development of guidance document and programmatic tools for planning and implementing strategies to scale up PPFP.

### Limitations

First, although a comprehensive literature search was conducted and a meticulous screening process was performed, yet the possibility of unpublished work always exists. Second, the adoption of clear criteria for what constitutes a standalone scaling up strategy was a major challenge. Each theme of scaling up PPFP contains a diversity of possible processes, content, and operational environments. Because these variables are often not controlled across studies, it is difficult to rigorously determine the situations in which specific strategies work best. Finally, information regarding the processes of scaling up strategies were not described in sufficiently informative details.

## Conclusions

Post pregnancy family planning can be scaled up using different strategies across a range of settings. This scale up appears to improve the uptake and utilization of post pregnancy contraceptive use. Programs striving to achieve a high impact need to overcome the most critical identified barriers namely those related to counselling and those related to integrating PPFP with postpartum or post-abortion care.

## Supplementary Information

**Additional file 1: Supplementary File 1.** The SURE Framework. **Supplementary File 2.** Data extraction form: Scaling up post pregnancy FP. **Supplementary File 3.** Mixed Methods Appraisal Tool (MMAT).

## Data Availability

All data generated or analysed during this study are included in this published article and its supplementary information files.

## Abbreviations

CERQual: Confidence in the Evidence from Reviews of Qualitative research
EPOC: Effective Practice and Organization of Care
FP: Family Planning
GRADE: Grading of Recommendations Assessment, Development, and Evaluation
HSBB: Health System Building Blocks
JBI: Joanna Briggs Institute
LMIC: Low- and middle-income countries
MMAT: Mixed Methods Appraisal Tool
MMSR: Mixed methods systematic reviews
PPFP: Post Pregnancy Family Planning
PRISMA: Preferred Reporting Items for Systematic Reviews and Meta-Analyses
SURE: Supporting the Use of Research Evidence
WHO: World Health Organization
