# Supplementary material for: Implementation strategies, facilitators, and barriers to scaling up and sustaining post pregnancy family planning, a mixed-methods systematic review
Source: BMC Womens Health. 2023 Jul 19;23:379. doi: 10.1186/s12905-023-02518-6 (PMC10357879; doi:10.1186/s12905-023-02518-6)
Supplement: Supplementary file 1 — Additional file 1: Supplementary File 1. The SURE Framework. Supplementary File 2. Data extraction form: Scaling up post pregnancy FP. Supplementary File 3. Mixed Methods Appraisal Tool (MMAT). [file 12905_2023_2518_MOESM1_ESM.docx]

**Supplementary File 1**

**The SURE Framework**

| **Level** | **Factors affecting implementation** |
| --- | --- |
| Recipients of care | Knowledge and skills; Attitudes regarding program acceptability, appropriateness, and credibility; Motivation to change or adopt new behavior |
| Providers of care | Knowledge and skills; Attitudes regarding program acceptability, appropriateness, and credibility; Motivation to change or adopt new behavior |
| Other stakeholders (including other healthcare providers, community health committees, community leaders, programme managers, donors, policymakers and opinion leaders) | Knowledge and skills; Attitudes regarding program acceptability, appropriateness, and credibility; Motivation to change or adopt new behavior |
| Health system constraints | Accessibility of care; Financial resources; Human resources; Educational and training system, including recruitment and selection; Clinical supervision, support structures and guidelines; Internal communication; External communication; Allocation of authority; Accountability; Community participation; Management and/or leadership; Information systems; Scale of private sector care; Facilities; Patient flow processes; Procurement and distribution systems; Incentives; Bureaucracy; Relationship with norms and standards. |
| Social and political constraints | Ideology; Governance; Short‐term thinking; Contracts; Legislation or regulation; Donor policies; Influential people; Corruption; Political stability and commitment. |

Supplementary File 2

Data extraction form: Scaling up post pregnancy FP

| Reviewer | Click or tap here to enter text. | Data extraction date: | Click or tap to enter a date. |
| --- | --- | --- | --- |

Meta-data

| First Author | Click or tap here to enter text. | Publication date: | Click or tap to enter a date. |
| --- | --- | --- | --- |
| Source: | Choose an item. | Click or tap here to enter text. | |
| Unique ID | Choose an item. | Click or tap here to enter text. | |
| Country of study | Click or tap here to enter text. | Country income level | Choose an item. |
| Funding source | Click or tap here to enter text. |  | |

Methods

| Type of study | Choose an item. |
| --- | --- |
| Methodology: | Click or tap here to enter text. |
| Phenomena of interest: | Click or tap here to enter text. |
| Number of participants: | Click or tap here to enter text. |

Characteristics of participants:

Click or tap here to enter text.

Scaling up component(s):

Click or tap here to enter text.

Setting and other context-related information:

Click or tap here to enter text.

Outcomes or findings of significance to the review objectives

Click or tap here to enter text.

Barriers

Click or tap here to enter text.

Facilitators

Click or tap here to enter text.

Author’s conclusion

Click or tap here to enter text.

Reviewer’s comments

Click or tap here to enter text.

Supplementary File 3

Mixed Methods Appraisal Tool (MMAT)

| Category of study designs | Methodological quality criteria |  | | Responses | |
| --- | --- | --- | --- | --- | --- |
|  |  | Yes | No | Can’t tell | Comments |
| Screening questions (for all types) | S1. Are there clear research questions? |  |  |  |  |
|  | S2. Do the collected data allow to address the research questions? |  |  |  |  |
|  | Further appraisal may not be feasible or appropriate when the answer is ‘No’ or ‘Can’t tell’ to one or both screening questions. | | |  | |
| 1. Qualitative | 1.1. Is the qualitative approach appropriate to answer the research question? |  |  |  |  |
|  | 1.2. Are the qualitative data collection methods adequate to address the research question? |  |  |  |  |
|  | 1.3. Are the findings adequately derived from the data? |  |  |  |  |
|  | 1.4. Is the interpretation of results sufficiently substantiated by data? |  |  |  |  |
|  | 1.5. Is there coherence between qualitative data sources, collection, analysis and interpretation? |  |  |  |  |
| 2. Quantitative  randomized controlled  trials | 2.1. Is randomization appropriately performed? |  |  |  |  |
|  | 2.2. Are the groups comparable at baseline? |  |  |  |  |
|  | 2.3. Are there complete outcome data? |  |  |  |  |
|  | 2.4. Are outcome assessors blinded to the intervention provided? |  |  |  |  |
|  | 2.5 Did the participants adhere to the assigned intervention? |  |  |  |  |
| 3. Quantitative nonrandomized | 3.1. Are the participants representative of the target population? |  |  |  |  |
|  | 3.2. Are measurements appropriate regarding both the outcome and intervention (or exposure)? |  |  |  |  |
|  | 3.3. Are there complete outcome data? |  |  |  |  |
|  | 3.4. Are the confounders accounted for in the design and analysis? |  |  |  |  |
|  | 3.5. During the study period, is the intervention administered (or exposure occurred) as intended? |  |  |  |  |
| 4. Quantitative descriptive | 4.1. Is the sampling strategy relevant to address the research question? |  |  |  |  |
|  | 4.2. Is the sample representative of the target population? |  |  |  |  |
|  | 4.3. Are the measurements appropriate? |  |  |  |  |
|  | 4.4. Is the risk of nonresponse bias low? |  |  |  |  |
|  | 4.5. Is the statistical analysis appropriate to answer the research question? |  |  |  |  |
| 5. Mixed methods | 5.1. Is there an adequate rationale for using a mixed methods design to address the research question? |  |  |  |  |
|  | 5.2. Are the different components of the study effectively integrated to answer the research question? |  |  |  |  |
|  | 5.3. Are the outputs of the integration of qualitative and quantitative components adequately interpreted? |  |  |  |  |
|  | 5.4. Are divergences and inconsistencies between quantitative and qualitative results adequately addressed? |  |  |  |  |
|  | 5.5. Do the different components of the study adhere to the quality criteria of each tradition of the methods involved? |  |  |  |  |

2
